# CRISP method with flipped classroom approach in ECG teaching of arrhythmia for trainee nurses: a randomized controlled study

**DOI:** 10.1186/s12909-022-03932-4

**Published:** 2022-12-07

**Authors:** Heling Wen, Min Hong, Fuli Chen, Xiaoyan Jiang, Rui Zhang, Jianhui Zeng, Lei Peng, Yu Chen

**Affiliations:** 1 Department of Cardiology, Sichuan Academy of Medical Science &Sichuan Provincial People’s Hospital, University of Electronic Science and Technology of China, 610072 Chengdu, China; 2 Center for Health Management, Sichuan Provincial People‘s Hospital, Sichuan Academy of Medical Science, University of Electronic Science and Technology of China, Chengdu, China; 3 Department of Cardiovascular Surgery, The Seventh People’s Hospital of Chengdu, Chengdu, China; 4 Department of Cardiology, The Affiliated Hospital of North Sichuan Medical College, Nanchong, China; 5 Department of Nephrology, Sichuan Academy of Medical Science &Sichuan Provincial People’s Hospital, University of Electronic Science and Technology of China, 610072 Chengdu, China

**Keywords:** CRISP method, Flipped classroom, Lecture-based learning, ECG interpretation, Nurses

## Abstract

**Background:**

This study aimed to explored the effects of the Cardiac Rhythm Identification for Simple People (CRISP) method with flipped classroom approach for arrhythmia interpretation in electrocardiogram (ECG) by trainee nurses.

**Methods:**

A total of 120 trainee nurses were enrolled and randomly divided into the experimental group and the control group using lecture-based learning method. We observed the effects of the two methods in ECG interpretation training and investigated the students’ attitudes toward the teaching practices.

**Results:**

After training, the ECG test scores in the experimental group were significantly higher than that of the control group. Six months later, the ECG test scores of the experimental group was still higher. Self-learning enthusiasm, understanding of teaching content, satisfaction of teaching mode, satisfaction of teaching effectiveness, and interest in learning ECG were significantly higher in the experimental group.

**Conclusion:**

CRISP method with flipped classroom approach is a new and effective mode worth trying in ECG teaching for trainee nurses.

**Supplementary Information:**

The online version contains supplementary material available at 10.1186/s12909-022-03932-4.

## Introduction

Electrocardiograms (ECGs) are one of the most common and essential tools in diagnosing cardiovascular diseases, especially for the rapid diagnosis of malignant arrhythmia and acute coronary syndrome [1]. In the field of cardiology (including cardiac care unit and cardiac catheterization laboratory), an ECG is critical for high-risk patients to make medical decisions. High-risk ECG patterns are frequently misdiagnosed, or are often missed, leading to adverse patient prognosis [2].

Recent literature has shown that the ECG interpretation skills of medical staff are highly variable. It has been suggested that medical education programs should ensure that no trainee has missed training in electrocardiographic emergencies [3]. Nurses are often the first discoverers of in-patient hospital cardiac arrests, and they can identify and deal with lethal arrhythmia in time after finding these life-threatening arrhythmias. Rapid and accurate interpretation ability of cardiac arrhythmias by nurses is vital to promoting positive patient outcomes [4]. ECG interpretation is integrated into undergraduate nursing education in several countries [5]. However, limited class hours, complicated teaching content, and weak basic knowledge of ECG theory bring great difficulties to ECG teaching and learning in the nursing specialty. It has been shown that nurses lack competence in recognizing cardiac rhythms, and medical educators need to explore new strategies to improve nurses’ learning in ECG interpretation [4, 5].

Traditional lecture-based learning (LBL) is still the most commonly adopted method in ECG teaching at present. However, ECG interpretation requires a high level of application [6]. Students are passive during LBL and have almost no opportunity to develop independent thinking and problem-solving skills [7, 8]. LBL thus may not be effective for nurses’ ECG teaching. The flipped classroom approach is a relatively new teaching model that readjusts the time inside and outside the classroom and transfers the leading role from teachers to students [8–10]. It has the potential to maximize the use of classroom time for promoting the application of knowledge, which meets the specific demands of ECG teaching. The current study demonstrated that the flipped classroom approach can enhance the learning engagement of medical students and improve their ECG self-learning capabilities [11]. Several recent systematic reviews have shown that, compared to traditional teaching methods, the flipped classroom approach was well received and preferred by medical students [12–14]. This method can therefore yield a significant improvement in nurses’ ECG learning [12]. The CRISP (Cardiac Rhythm Identification for Simple People) method is an innovative algorithm designed to help nurses rapidly interpret ECGs [15, 16]. It has been shown that the CRISP method for interpreting primary cardiac arrhythmia by nurses is effective, simple, easy. Nurses’ competence in ECG interpretation, especially for accurate assessment of fatal arrhythmias, increased significantly after training [16].

Our study hypothesized that a teaching method that combined the virtues of the CRISP method and a flipped classroom approach could better achieve the goal of promoting effective ECG teaching for trainee nurses. By comparing it with LBL, this study sought to observe the effects of the two different teaching methods in ECG interpretation training and investigated the students’ attitudes toward this teaching practice. To our knowledge, there have been no studies that have analyzed the outcome of this combined pedagogy in trainee nurses’ ECG teaching.

## Methods

This was a randomized controlled trial study with two groups including three ECG interpretation tests and a quantitative questionnaire survey. A total of 120 trainee nurses in the Department of Cardiology were enrolled. All of them were female, aged 19–25 years. All the trainee nurses were graduates of nursing faculties and had passed the nursing license. None of them had received systematic training in ECG interpretation before. Random grouping was applied using SPSS 26.0 (SPSS Inc., Chicago, IL, USA). The randomization code was generated and sealed in the envelopes. Before the study, a sealed envelope containing the randomization code was opened for every trainee nurse. Enrolled trainee nurses were randomly assigned to receive either the flipped classroom with the CRISP method (experimental group) or an LBL (control group) training. These procedures were conducted by a teacher who was not involved in this study. The researchers were only made aware of the group allocation after the randomization was performed. The class was held when the trainee nurses were in the second month of a one-year cardiology training program. The one-year cardiology training program aims to prepare the nurses to become competent, caring, and professional nurses who can work in a cardiac catheterization laboratory or cardiac care unit. All of the trainee nurses were randomly divided into the experimental and control groups, with 60 nurses in each group.

**Fig. 1:**
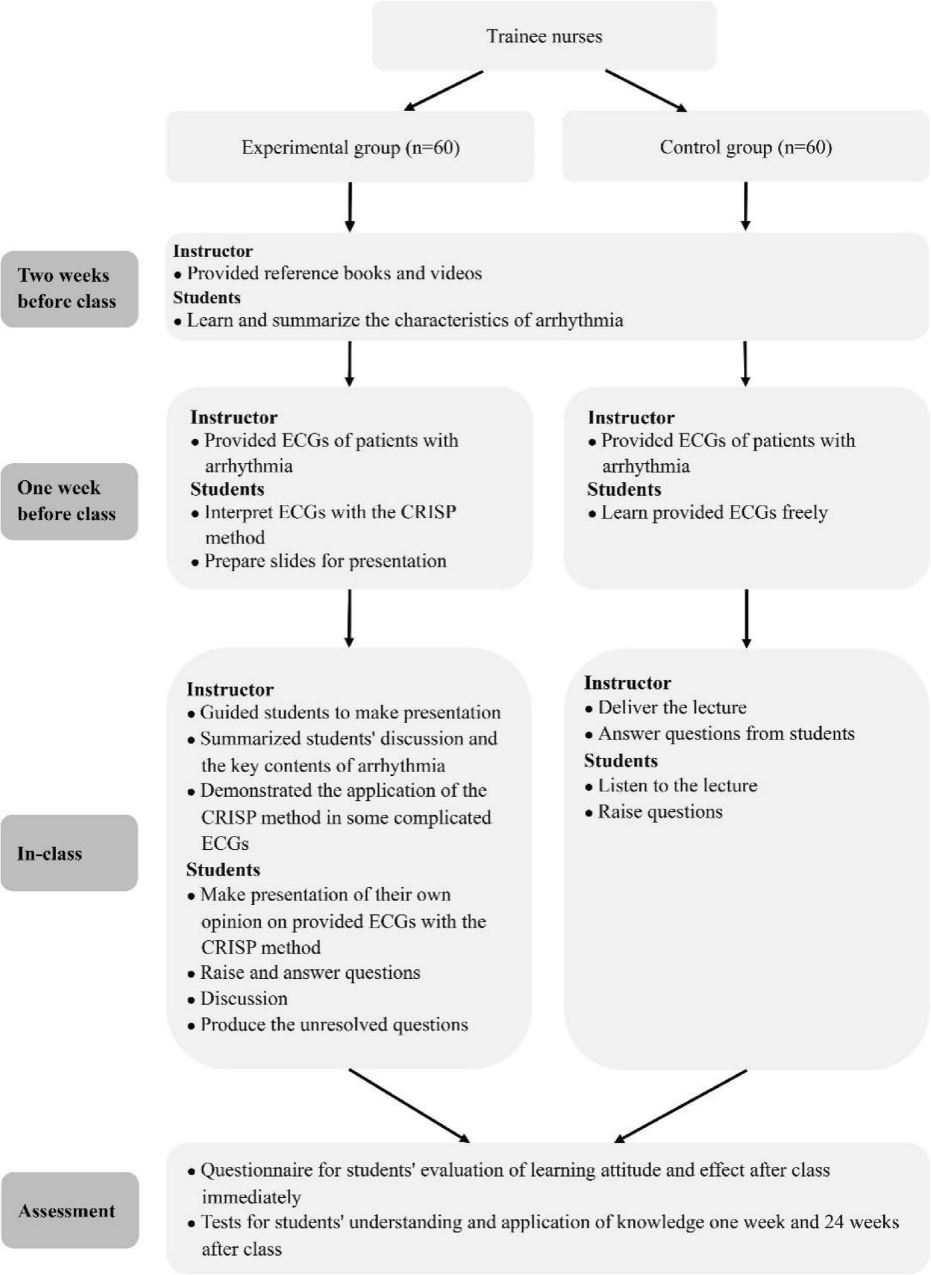
Schematic demonstration of the process of teaching activities. Control group: traditional LBL method; Experimental group: flipped classroom combined with the CRISP method

This randomized controlled trial study was conducted in the training and research hospital during the academic years of 2018–2020. About 40 trainee nurses were enrolled in each academic year, and they were divided into the experimental and control groups, which meant there were approximately 20 trainee nurses in each group. Each class was held face-to-face with about 20 students at one time. This arrhythmia education course included four sessions with 45 min per session and lasted for two weeks. The two groups received the same reference books and the same syllabus. The trainee nurses in the two groups were taught by the same faculty members, including one teacher and three teaching assistants. The teacher was a cardiovascular physician, who taught all courses; he was also an experienced teacher of cardiology and diagnostics in the medical college. The study was carried out according to the flowchart displayed in Fig. 1.

The flipped classroom with the CRISP method was conducted in this group as follows. Two weeks before class, students were provided with the designated professional reference books and ECG learning videos to learn and summarize arrhythmia characteristics independently. One week before class, students were provided with 38 ECGs of patients with various arrhythmia or normal rhythms accumulated in clinical practice anonymously consistent with the syllabus. The ECGs included normal sinus rhythm, premature ventricular contractions, premature junctional contractions, premature atrial contractions, sinus tachycardia, supraventricular tachycardia, atrial tachycardia, atrial fibrillation, atrial flutter, ventricular fibrillation, ventricular tachycardia, sinus bradycardia, sinus asystole, junctional escape rhythm, ventricular escape rhythm, first-degree atrioventricular block, second-degree atrioventricular block–Mobitz I, second-degree atrioventricular block–Mobitz II, and third-degree atrioventricular block. Every disease or normal condition was represented by two ECGs.

Students were required to interpret ECGs with the CRISP method and prepare slides for their presentations. During classroom time, the teacher guided students to report their own opinion of those ECGs in their slides during the classroom phase. Moreover, all students were encouraged to ask questions or debate. This stage lasted approximately 30 min. Finally, teachers summarized the advantages and disadvantages of students’ discussions, demonstrated the application of the CRISP method in certain complicated ECGs, and briefly outlined the critical contents of various arrhythmia. This stage lasted approximately 15 min. After class, the students were free to learn based on their preferences.

**Fig. 2:**
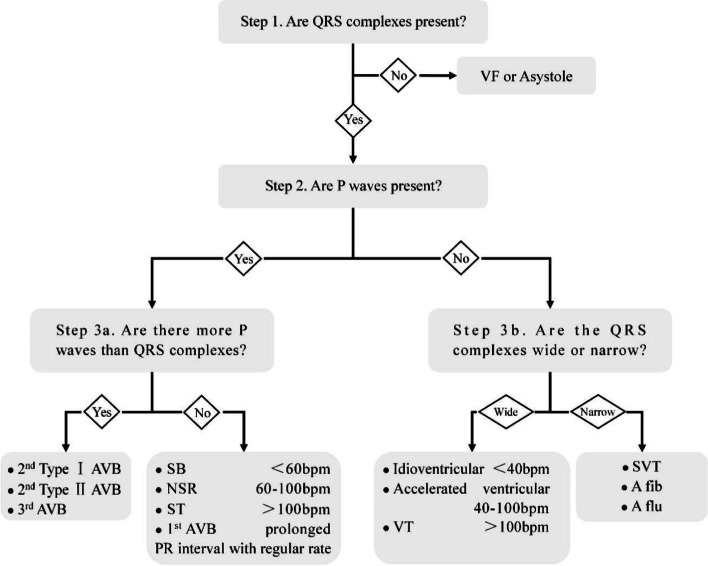
Summary of the CRISP algorithm. VF = ventricular fibrillation; 1st AVB = first-degree atrioventricular block; 2nd Type I AVB = second-degree atrioventricular block - Mobitz I; 2nd Type II AVB = second-degree atrioventricular block - Mobitz II; 3rd AVB = third-degree atrioventricular block; SB = sinus bradycardia; NSR = normal sinus rhythm; ST = sinus tachycardia; VT = ventricular tachycardia; SVT = supraventricular tachycardia; A fib = atrial fibrillation; A flu = atrial flutter

Using the CRISP method, in summary, to interpret ECGs of patients with various arrhythmia, the students asked and answered the following questions: (1) Are QRS complexes present? (2) Are P waves present? 3a. P waves are present. Are there more P waves than QRS complexes? 3b. No P Waves are present. Are the QRS complexes wide or narrow? 4. What is the appropriate treatment for this patient? A brief summary of the CRISP algorithm is shown in Fig. 2 [15, 16].

The LBL method was conducted in the control group as follows. Two weeks before class, students were provided with the same books and ECG teaching videos as the experimental group. One week before class, students were provided with the same patient ECGs as in the experimental group. During the course, teachers delivered a 40-minute lecture to explain various arrhythmia (including ECG characteristics and treatment) followed by approximately 5 min for asking and answering questions. Students in this group were not given the CRISP method to learn about ECGs, and the learning happened through lectures and textbooks. Before and after class, the students were free to learn based on their preferences.

To assess the students’ understanding and application of knowledge, the two groups had the same examination. An ECG interpretation test was adopted before training, one week and 24 weeks after training. The questions in the three tests were assessed to ensure consistency in difficulty levels by two different teachers. Each test included 20 ECGs of arrhythmia, with 5 points for each question. The total score was 100 points and the test time was 40 min. A set of examination questions has been included in the Supplemental Materials (Fig S1-S4).

To assess the students’ evaluation of learning attitudes and effect, a questionnaire survey was adopted at the end of the course, including self-learning enthusiasm, an increase of study load, systematization of teaching content, understanding of teaching content, satisfaction of teaching mode, satisfaction of teaching effectiveness and interest in learning about ECGs (Table S1). The 5-level Likert scoring method was adopted for each question, with 5 points for very satisfied/strongly agreed, 4 points for satisfied/agreed, 3 points for neutral, 2 points for dissatisfied/disagreed and 1 point for very dissatisfied/strongly disagreed [17].

The ethics committee approved the study. Before enrolment, all participants provided written consent, wherein they were informed that they could freely withdraw from the study. This study was conducted in accordance with the Declaration of Helsinki (2013).

### Statistical analysis

Data normality was evaluated using the Shapiro–Wilk test. Data were presented as mean ± standard deviations (SDs) or median (interquartile range, IQR) values, as appropriate according to data distribution. Ages were compared with the Mann–Whitney *U* test. ECG test scores before training of the two groups were compared by a *t*-test. For the analysis of the ECG test scores at different time points, comparisons were analyzed using analysis of variance (ANOVA) for repeated measures. For the 5-level Likert scores of students’ evaluation of learning attitudes and effect, the Mann–Whitney *U* test was applied. Statistical analyses were conducted in SPSS 26.0 (SPSS Inc., Chicago, USA). All tests were two-tailed, and significance was set at *p* < 0.05.

## Results

### Baseline of participants

A total of 120 trainee nurses were enrolled in this study. They had similar educational backgrounds. There was no significant difference in age and ECG test scores before training between the experimental and control groups (Table 1).

**Table 1:** Baseline of enrolled trainee nurses

Characteristics	Control Group (*n* = 60)	Experimental Group (*n* = 60)	z/t	*p*
**Age (Yrs)**	22.0 (21.0–23.0)	22.0 (21.0–23.0)	0.276	0.783^a^
**ECG test scores before training**	59.00 ± 7.18	60.43 ± 7.75	1.051	0.295^b^

^a^The two groups were compared using a Mann–Whitney *U* test

^b^The two groups were compared using an independent sample *t*-test

### Comparison of ECG test scores

**Fig. 3:**
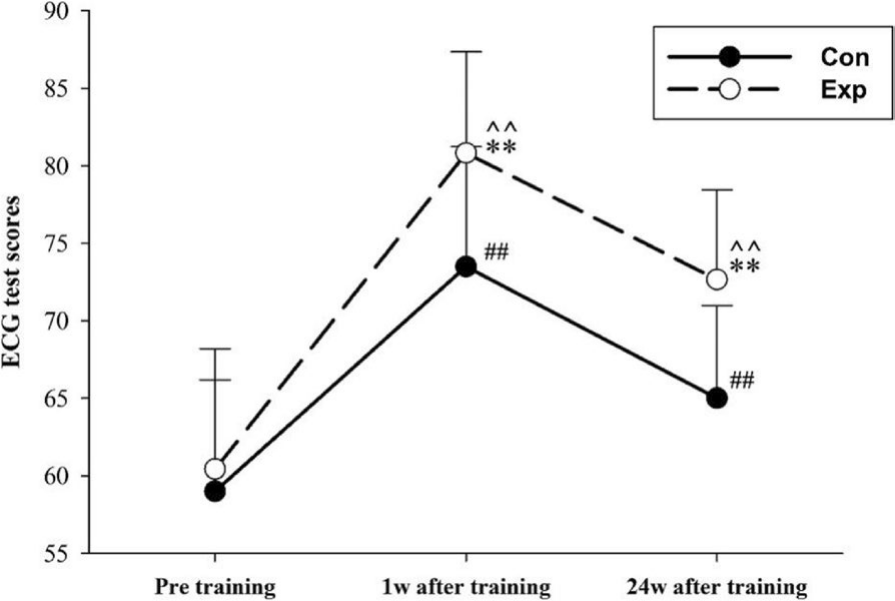
ECG test scores of Con (*n* = 60) and Exp (*n* = 60) groups at different time points. Con: control group with the traditional LBL method, Exp: experimental group of the flipped classroom combined with the CRISP method. Data are presented as mean ± standard deviation (SD); ** *p* < 0.001 vs. pre-training in Exp, ## *p* < 0.005 vs. pre-training in Con, ^^ *p* < 0.001 vs. Con at one week after training and 24 weeks after training

The ECG test scores before training were 59.00 ± 7.18 and 60.43 ± 7.75 in the control and experimental groups, respectively. One week after training, the ECG test score of the experimental group was significantly higher than that of the control group (80.83 ± 6.52 vs. 73.50 ± 7.72, *p* < 0.001). Twenty-four weeks after training, the ECG test scores of the two groups significantly decreased, but the score of the experimental group was still significantly higher (72.67 ± 5.78 vs. 65.00 ± 5.97, *p* < 0.001). The ECG test scores one week and 24 weeks after training of both groups increased notably compared to pre-training scores (*p* < 0.001) (Fig. 3).

### Comparison of students’ attitudes

**Fig. 4:**
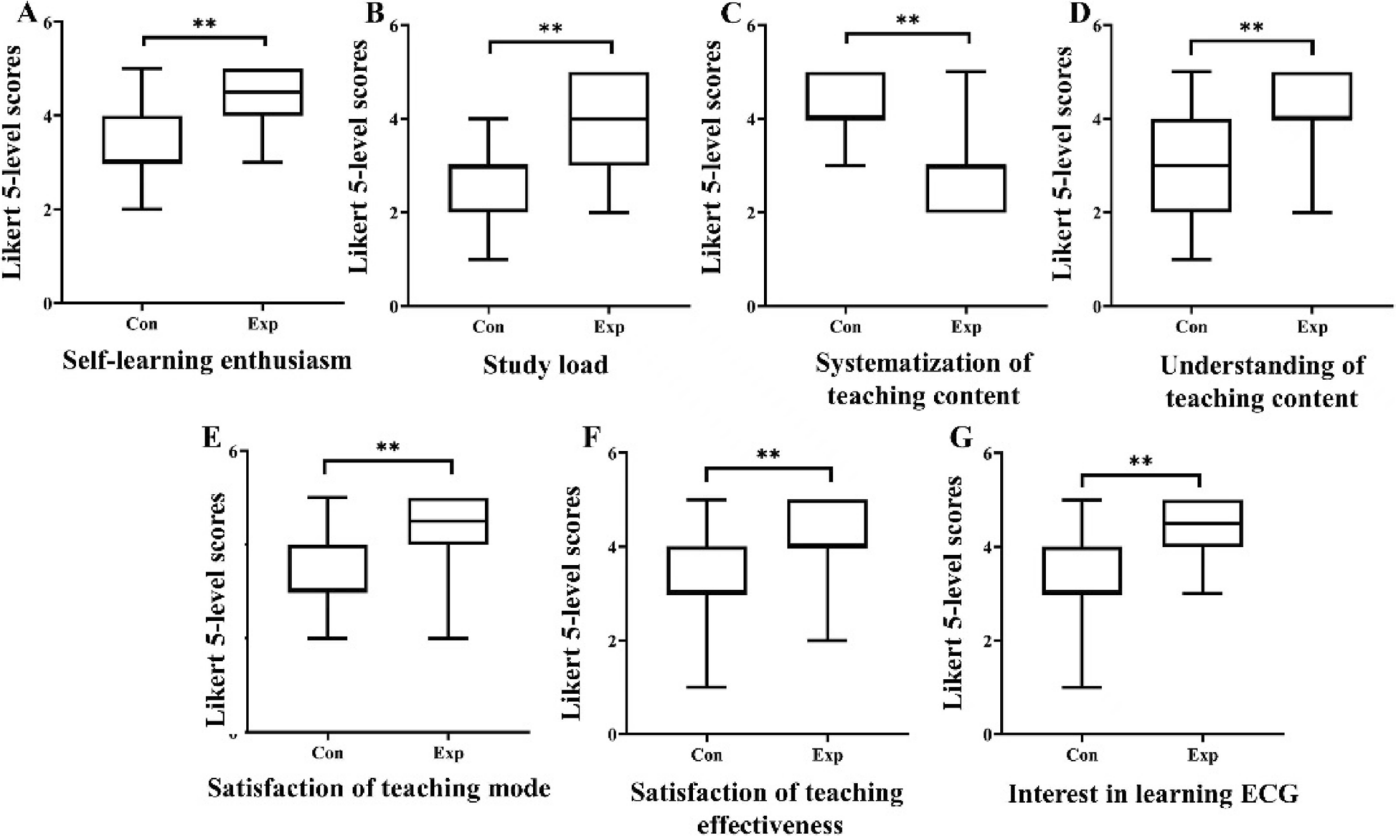
Five-level Likert scores of students’ attitudes in Con (*n* = 60) and Exp (*n* = 60) groups. **A** self-learning enthusiasm; **B** study load; **C** systematization of teaching content; **D** understanding of teaching content; **E** satisfaction of teaching mode; **F** satisfaction of teaching effect; **G** interests in continuing to learn about ECGs. Con: control group with the traditional LBL method, Exp: experimental group of the flipped classroom combined with the CRISP method. ** *p* < 0.001 Con vs. Exp

A total of 120 questionnaires were sent out and recovered with a recovery rate of 100%. Compared with the control group, self-learning enthusiasm, understanding of teaching content, satisfaction of teaching mode, satisfaction of teaching effectiveness, and interest in learning ECG in the experimental group were significantly higher than the control group (*p* < 0.001). Meanwhile, it is worth noting that the majority of students in the experimental group indicated an increase in study load and a lack of systematization of teaching content (*p* < 0.001) (Fig. 4).

## Discussion

It has been demonstrated that nurses’ early recognition and interpretation of ECG patterns correctly can enhance the quality of care and provide improved outcomes for patients [18, 19]. Therefore, improvement in nurses’ knowledge and interpretation of ECGs has become a necessary task in ECG teaching. In China, the curriculum for ECG theory and practice in the nursing specialty is significantly less emphasized than that of the clinical specialty. To most students in the nursing specialty, it is challenging to use abstract and complicated knowledge in working out an ECG diagnosis. How to simplify abstract knowledge and promote their understanding of ECG patterns may be more important than the improvement of lectures and textbooks.

The CRISP method is an algorithm designed to enable nurses to interpret ECGs rapidly [15]. Compared with traditional ECG analysis methods, it only takes three steps to identify the cardiac rhythm, then provides sufficient information for the risk stratification of arrhythmia. Using the CRISP method, it became easier to master ECG interpretations for trainee nurses, further inspiring students’ learning interests in ECGs. Meanwhile, the CRISP strategy did not increase the overall class hours; it can solve the difficulties imposed by the limitations in class hours, as these limitations have been problematic in ECG teaching for nurses. Using the CRISP method, the nurses were most successful with questions about fatal arrhythmias in the test after education [16]. However, the CRISP method also has some limitations. The participants in the study still had difficulty recognizing heart blocks after the CRISP method program [16]. The CRISP method is highly simplified, and it is difficult to provide more accurate rhythm descriptions and more precise identification, especially for complex arrhythmias [16].We need to further enrich the CRISP method to improve the effect of education on ECG interpretation by nurses.

The flipped classroom approach is a relatively new student-centered teaching mode, which is more suitable for the requirements of modern education [13, 20, 21]. It has been shown that implementation of the flipped classroom approach can allow students to learn independently in advance [22, 23]. They can acquire basic knowledge of arrhythmias, raise questions, and play leading roles in the classroom. During the limited classroom time, it is more important to improve ECG diagnosis capability than to deliver knowledge. The students in the flipped classroom scored significantly higher in the ECG test than the control group [11]. The traditional LBL method is considered a teacher-oriented educational mode. A systemic theoretical lecture is indeed the most efficient way to deliver information; however, LBL may not be suitable for high-grade medical students, who need to cultivate high-level cognitive learning skills, such as analytical thinking and problem solving. The teaching tools are generally slides and textbooks, and the teaching pattern is lectures. Students passively receive the knowledge disseminated. Their participation and initiative are low, so the learning effect cannot be guaranteed.

According to our practice, the flipped classroom has some limitations. It requires much more time and effort for teachers and students compared to LBL. Given the vastness of the nursing curriculum, the implementation of a flipped classroom may reduce the time students spend on another curriculum elements. Simultaneously, the flipped classroom approach lacks systemic and comprehensive knowledge delivery to students due to its teaching characteristics. Furthermore, the flipped classroom approach requires students to have higher ability to implement self-learning and peer coaching. The students’ ability difference in self-learning and peer coaching can affect the learning outcome in the flipped classroom approach. Bossier et al. found that a flipped classroom approach did not necessarily improve students’ performance on examination questions compared with the lecture cohort in a pharmacotherapy oncology module [24], so further research is needed to determine the optimal teaching skills and application scenarios for the flipped classroom approach.

It is not possible that one single method or format of teaching is the most effective one for improving ECG interpretation skills [25]. ECG interpretation is difficult, and capability in this area requires effort to acquire and maintain. Nurse educators should carefully assess the effect of the flipped classroom with the CRISP method to enhance nursing students’ ECG interpretation skills and knowledge of cardiac arrhythmias. In this study, we observed how the use of the CRISP method with a flipped classroom approach affected the accuracy of ECG interpretation by trainee nurses. Our study demonstrated that this accuracy was remarkably higher in the experimental group than in the control group. In combination with the CRISP method, the flipped classroom approach achieved a shift from knowledge delivery to ECG diagnosis capability improvement which might promote teaching efficiency to the greatest extent. Our study further confirmed the CRISP method with a flipped classroom approach is an effective way to improve medical students’ interest in learning ECGs and their self-learning abilities. The test score gap between the two groups after training might be partially attributable to the characteristics of the two teaching methods. We thought the most important difference was that the experimental group focused on improving understanding of the core, difficult parts raised by the students themselves and promoting the derivation and application of knowledge. Our study also revealed that satisfaction with the teaching mode, satisfaction with teaching effectiveness, and interest in learning about ECG in the experimental group was significantly higher than in the control group. Our results were consistent with previous results that the flipped classroom can improve study interest and subjective initiative [26].

The cognitive activities can be categorized into six hierarchical levels according to Bloom’s taxonomy: remember, understand, apply, analyze, evaluate, and create. The assessment of high-level cognition in Bloom’s taxonomy (application and analysis) has been recommended to evaluate the effectiveness of the flipped classroom approach [27]. Our training focused on improving the ECG interpretation skills of nurses. Our test consisted of 20 ECGs, and aimed to examine the accuracy of ECG interpretation by trainee nurses. All the questions were considered as clinical analyses, so they tested the application and analytical skills of Bloom’s taxonomy. The test questions were not particularly relevant to students’ memory, understanding, assessment, and creativity. Our finding was consistent with the results of a previous study, which found higher levels of application and analysis, as these were significantly higher in the flipped classroom approach group than in the traditional teaching group [27].

Another vital point of ECG training is to maintain knowledge and capability for a long time. Six months later, the ECG interpretation test showed that the scores of the two groups decreased significantly, but the scores in the experimental group were still higher than that of the control group. It indicated that the teaching effect of the flipped classroom with the CRISP method was more long-lasting. Findings from a similar study have also demonstrated that the flipped classroom approach promotes long-term content retention [28]. Ming Ji et al. found that the flipped classroom approach significantly improved the long-term learning outcomes in pathophysiology, pathology, pharmacology diagnostics, and internal medicine compared with the control group [29] There are three possible reasons for this. First, the flipped classroom approach can enhance students’ lifelong learning skills, including the ability to acquire new knowledge [29] and stimulate their self-learning interests. It is particularly helpful for residual educational benefits over time [30]. Second, the flipped classroom approach can strengthen the understanding and application of the acquired knowledge and promote the students’ deep learning, resulting in enhancement of the students’ logical, analytical, and knowledge application skills [31]. Third, because the CRISP method is a simple and easy way to ensure accurate ECG interpretation by nurses, it is helpful for students to remember and use it over time [16].

## Limitations

This study has three main limitations. First, our study did not evaluate the students’ understanding and application of knowledge over a longer period of time. More time points (36-week and 48-week after class) should be set to investigate the longevity of the retained knowledge. Second, this study only was performed in trainee nurses. Further large-scale studies are needed to verify its effectiveness for residents. Third, our study did not investigate the effect of the CRISP method or flipped classroom approach in isolation on the arrhythmia interpretation accuracy by trainee nurses. In the future, further large-scale investigations are needed to identify the specific roles of these two approaches, thereby determining to what extent the educational effects were achieved using the CRISP method alone and to what extent they were achieved using the flipped classroom approach.

## Conclusion

The CRISP method may be an easy and simple way to enhance students’ clinical skills in the identification of arrhythmia in ECGs. The application of the flipped classroom, combined with the CRISP method, in the teaching of ECGs has an excellent effect on ECG interpretation capability, self-learning enthusiasm, understanding of teaching content, satisfaction of teaching mode, satisfaction of teaching effectiveness, and interest in learning for nursing students. It is a new and effective mode that needs to be further studied and promoted for more trainee nurses.

## Supplementary Information

**Additional file 1.**

## Data Availability

Please contact the corresponding author for data availability.
