# Supplementary material for: CRISP method with flipped classroom approach in ECG teaching of arrhythmia for trainee nurses: a randomized controlled study
Source: BMC Med Educ. 2022 Dec 7;22:850. doi: 10.1186/s12909-022-03932-4 (PMC9730600; doi:10.1186/s12909-022-03932-4)
Supplement: Supplementary file 1 — Additional file 1. [file 12909_2022_3932_MOESM1_ESM.docx]

**Supplemental Material**

**Supplemental figure legends**

Figure S1. Questions 1-5 in a set of ECG interpretation Test.


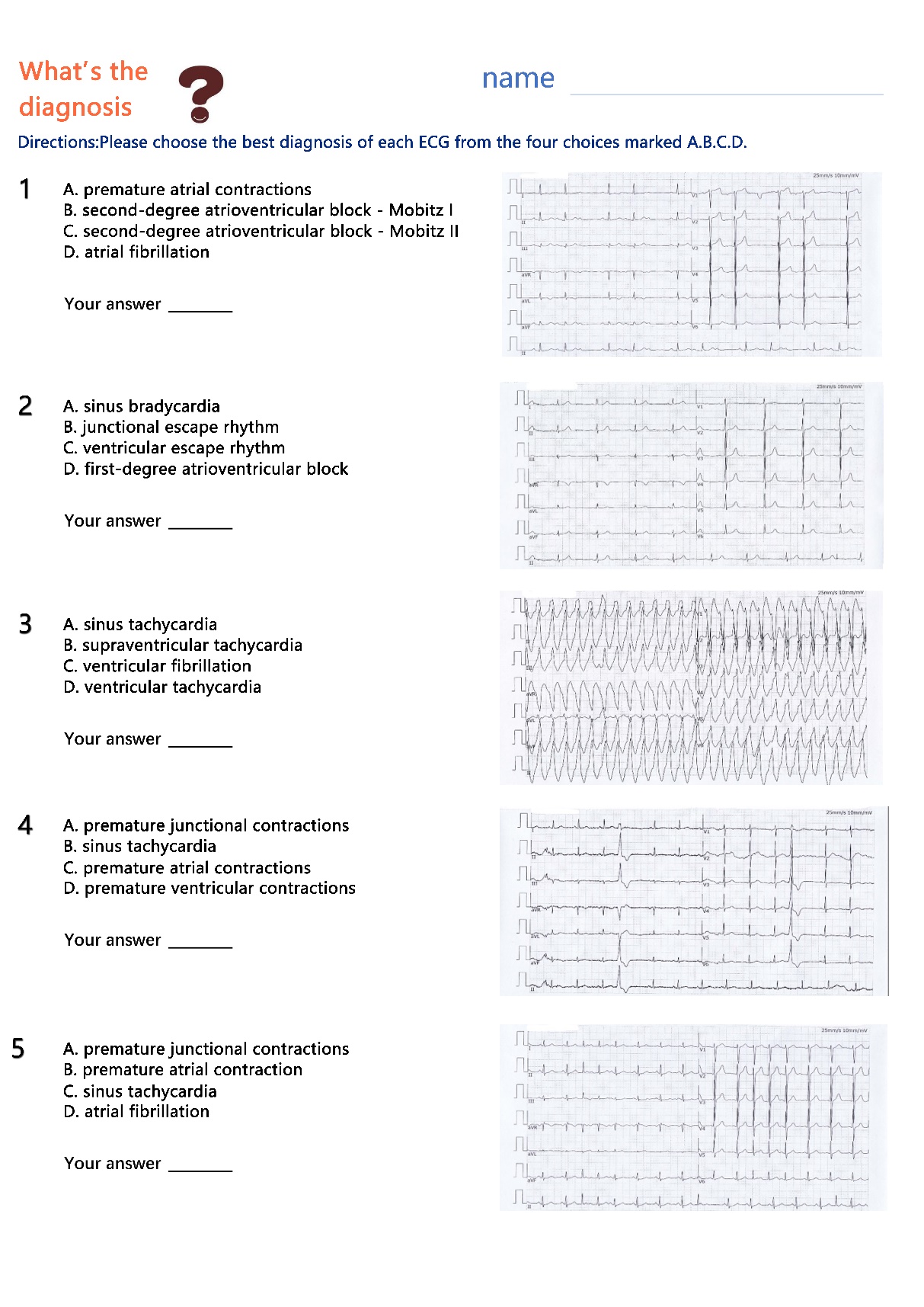


Figure S2. Questions 6-10 in a set of ECG interpretation Test.


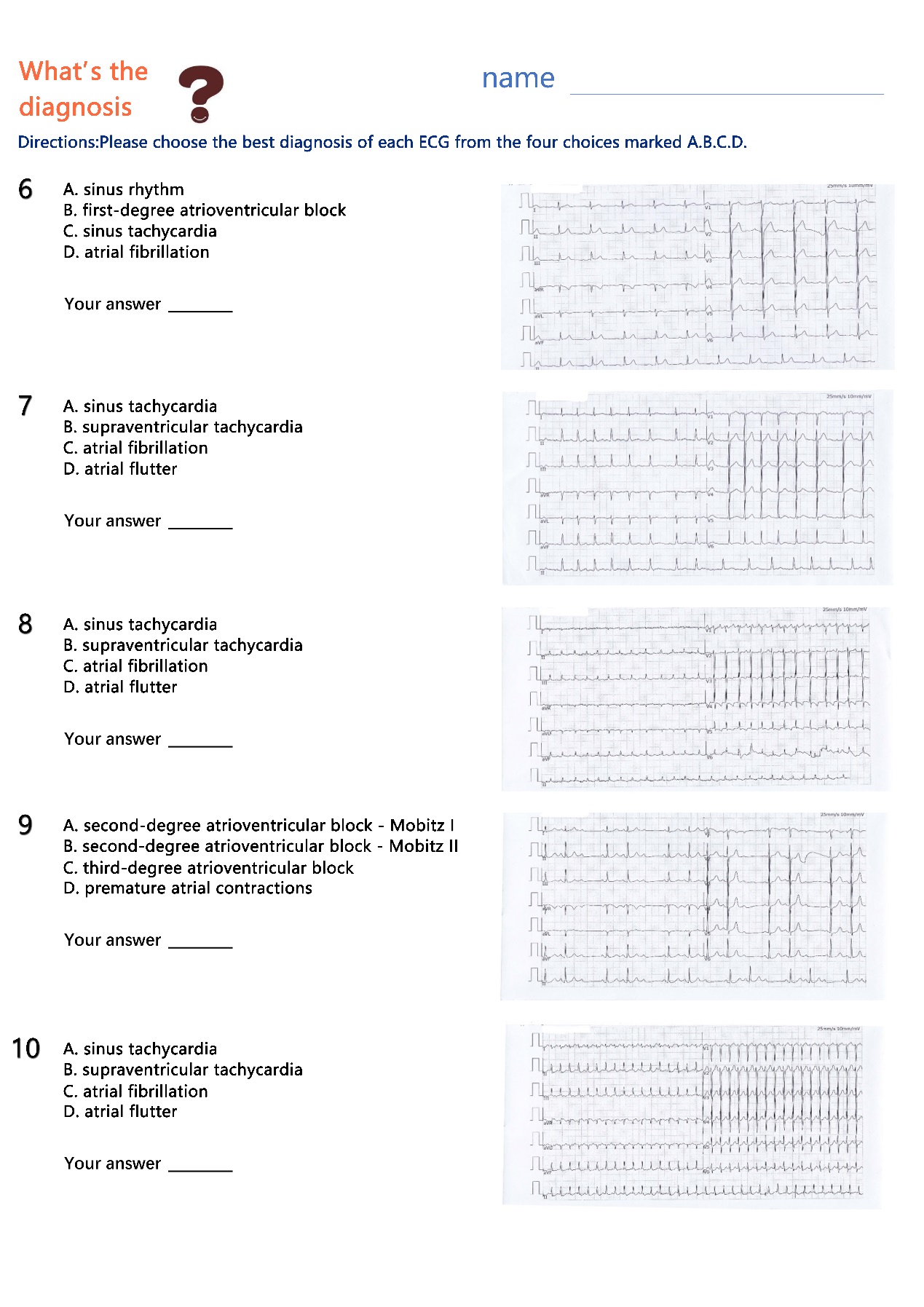


Figure S3. Questions 11-15 in a set of ECG interpretation Test.


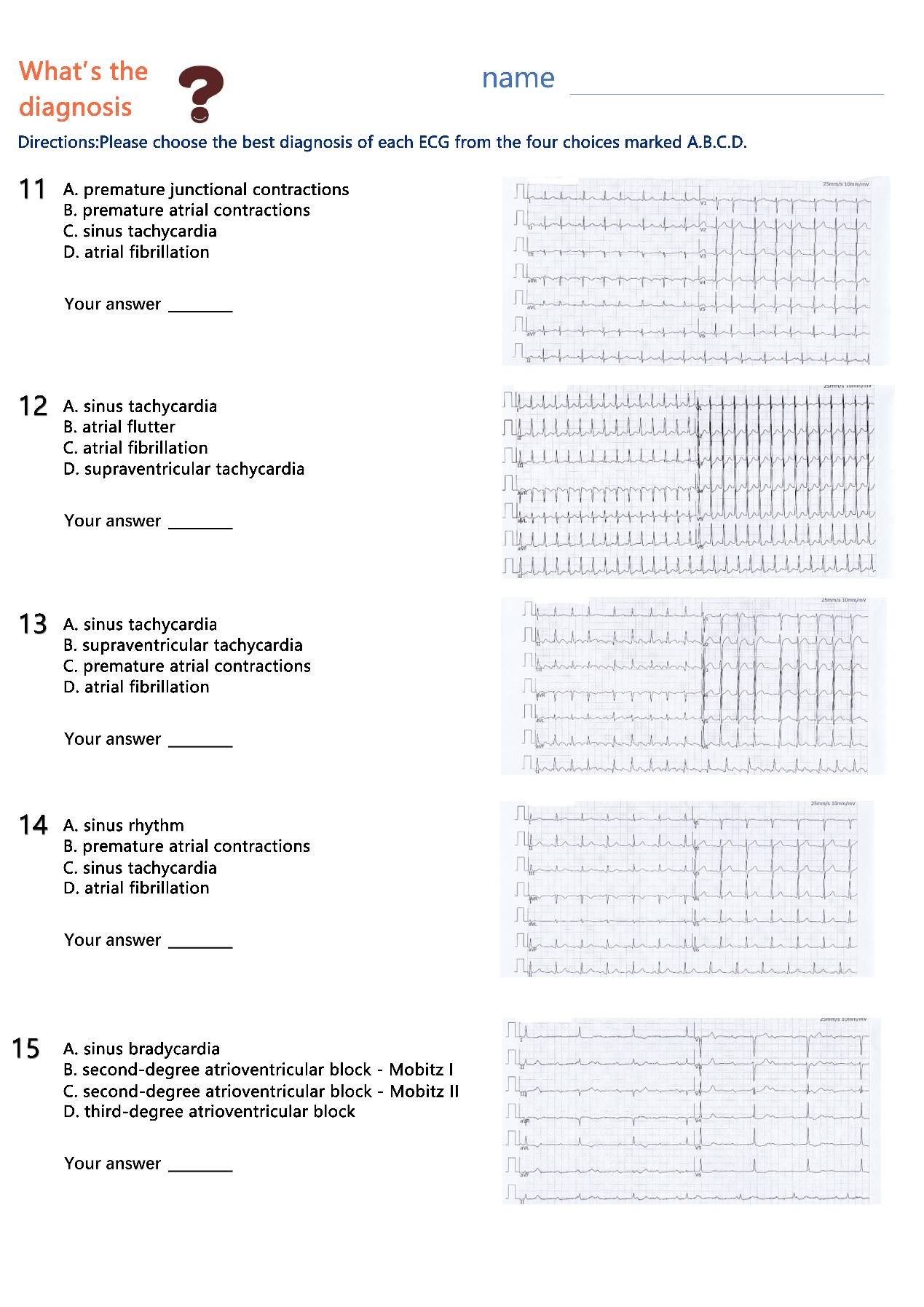


Figure S4. Questions 16-20 in a set of ECG interpretation Test.


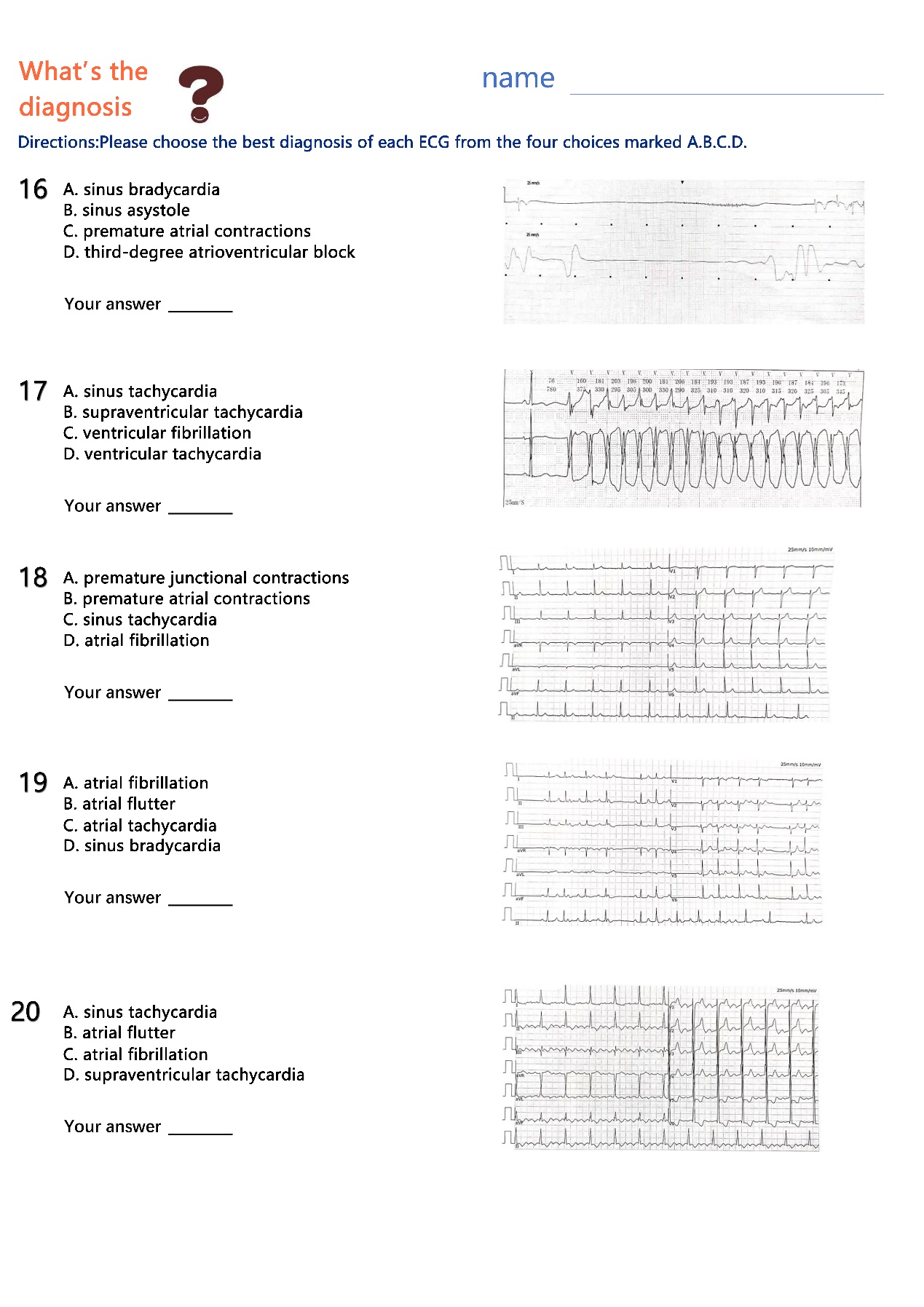


**Supplemental Table**

Table S1. Students’ attitudes towards the teaching modes

| Questions |
| --- |
| 1. This teaching mode increases your self-learning enthusiasm   1 strongly disagreed, 2 disagreed, 3 neutral, 4 agreed, 5 strongly agreed   1. This teaching mode increases your study load   1 strongly disagreed, 2 disagreed, 3 neutral, 4 agreed, 5 strongly agreed   1. This teaching mode has systematization of teaching content   1 strongly disagreed, 2 disagreed, 3 neutral, 4 agreed, 5 strongly agreed   1. This teaching mode was helpful in understanding of teaching content   1 strongly disagreed, 2 disagreed, 3 neutral, 4 agreed, 5 strongly agreed   1. Are you satisfied with this teaching mode   1 very dissatisfied, 2 dissatisfied, 3 neutral, 4 satisfied, 5 very satisfied   1. Are you satisfied with the teaching effect   1 very dissatisfied, 2 dissatisfied, 3 neutral, 4 satisfied, 5 very satisfied   1. This teaching mode increases your interests in continuing to learn about ECGs   1 strongly disagreed, 2 disagreed, 3 neutral, 4 agreed, 5 strongly agreed |
